# Randomized, wait-list–controlled pilot study of app-delivered mindfulness for patients reporting chronic pain

**DOI:** 10.1097/PR9.0000000000000924

**Published:** 2021-04-02

**Authors:** Jennifer S. Mascaro, Vinita Singh, Kathryn Wehrmeyer, Benjamin Scott, Justin Juan, Anne Marie McKenzie-Brown, Olabisi P. Lane, Carla Haack

**Affiliations:** Departments of a Family and Preventive Medicine,; b Anesthesiology, and; c Surgery, Emory University, Atlanta, GA, USA

**Keywords:** Mindfulness, Chronic pain, Meditation, Mobile Health

## Abstract

**Introduction:**

Chronic pain creates economic burden and exerts profound individual and societal harm. Mobile application (app)-delivered mindfulness meditation may be an important approach to self-management of chronic pain.

**Objectives:**

We examined the feasibility, acceptability, and impact of app-delivered mindfulness meditation on pain cognition and daily functioning among patients reporting chronic pain.

**Methods:**

We used a longitudinal, randomized, and wait-list–controlled design (NCT03495726) to evaluate changes in self-reported pain severity, pain catastrophizing, and social and physical functioning among participants randomized to 6 weeks of app-delivered mindfulness meditation, compared with participants randomized to a wait-list control group.

**Results:**

Although most participants randomized to the mindfulness group used the app at least once, fewer than half adhered to the instructed program. Participants who did not use the app scored higher on the helplessness component of pain catastrophizing at the start of the study and were less likely to have completed 4 years of college. Participants who reported feeling pressured to enroll in the study were also less likely to adhere to the intervention. Compared with participants randomized to wait-list, those in the mindfulness group reported significant improvements in social functioning, even after controlling for pain severity. Participants randomized to the mindfulness intervention also reported significant improvements in helplessness. App usage was not significantly correlated with changes in social functioning or helplessness scores.

**Conclusions:**

These results suggest that app-delivered mindfulness meditation is beneficial to patients with chronic pain. Identifying characteristics of patients who were adherent highlights important considerations for clinical settings.

## Abstract 2

App-delivered mindfulness improved social functioning and pain catastrophizing among patients with chronic pain. Patient characteristics predicted app engagement, highlighting important considerations for clinical settings.

## 1. Introduction

Chronic pain affects an estimated 11.2% of the U.S. population,^29^ costs the country approximately $635 billion per year in medical expenses and lost productivity,^15^ and has contributed to the rise in opioid addiction.^30^ New guidelines emphasizing non pharmacologic treatment options^35^ have encouraged practitioners to find alternative approaches to managing chronic pain, in addition to opioid prescribing.^12^

Preliminary studies indicate that mindfulness meditation holds promise for reducing the distress of chronic pain.^5,16,35^ Mindfulness training decreases the suffering that accompanies painful stimuli,^48,50^ an effect that seems in part to be orthogonal to opioid-dependent pain processing.^47^ Evidence indicates that mindfulness-based interventions (MBIs) may be effective for patients with chronic pain, reducing perceived pain and decreasing harmful psychological features often associated with chronic pain, such as depression and pain catastrophizing.^1,7,19,24,37^ In addition, emerging data support the role of MBIs for augmenting positive emotions and coping skills for patients experiencing chronic pain. Coping skills identified include increases in self-efficacy for managing pain, resulting in improved quality of life.^1,25^

Although mindfulness can be an effective approach to the self-management of chronic pain, most research to date has been conducted on either brief inductions of mindfulness^14,28,49^ or on more time-intensive and resource-intensive group-based interventions.^7,19^ The effectiveness of mindfulness delivered by smartphone applications (apps) for chronic pain management has not been well studied or characterized. However, Jamison et al. demonstrated that more than 90% of patients with chronic pain report a willingness to use a smartphone app daily for at least 6 months, establishing the potential feasibility of self-management of chronic pain using mindfulness apps.^21^ Moreover, app-delivered mindfulness is one of the resources most frequently recommended by health care providers for the self-management of chronic pain.^11^ Pain is one of the most common physical health diagnoses reported by users of mindfulness apps,^20^ highlighting the critical need for examining the effectiveness of app-delivered MBIs.

To this end, we examined the feasibility and effectiveness of app-delivered mindfulness meditation for patients experiencing chronic pain. Specifically, we aimed to evaluate the feasibility of using a smartphone app for mindfulness meditation and to identify the characteristics of patients who adhered to the mindfulness intervention. We also aimed to evaluate changes in pain severity, pain catastrophizing, and daily functioning among patients randomized to mindfulness, compared with patients randomized to a wait-list control group.

## 2. Methods

### 2.1. Participant characteristics

**Figure 1.**
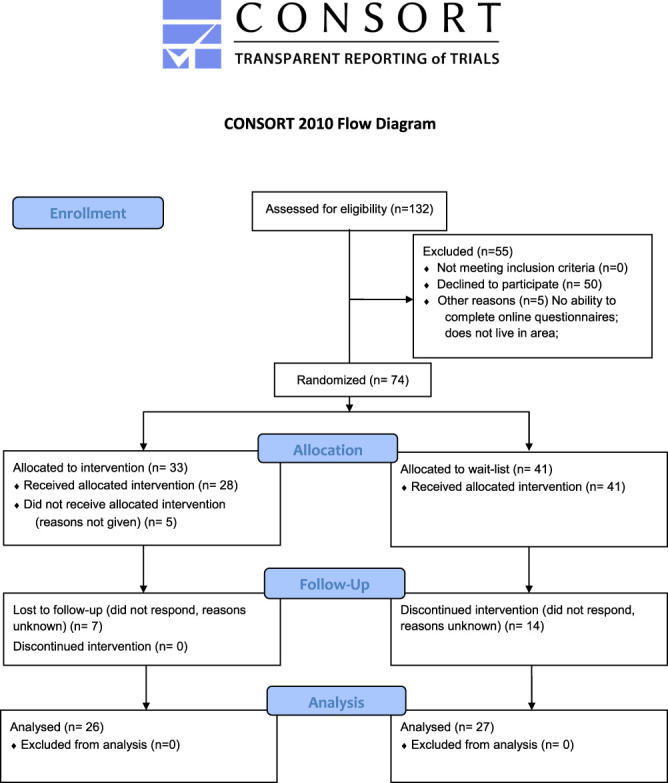
CONSORT flow diagram of the enrollment, allocation, follow-up, and assessment of the parallel randomization of patients into intervention and wait-list groups.

Participants were patients within the Emory Healthcare system and enrolled through in-person recruitment from the Emory Pain Center (Fig. 1). Recruitment methods included (1) in-person recruitment by a research coordinator during a patient's scheduled clinic visit, (2) physician referral from the Emory Pain Center, and (3) self-referral through ClinicalTrials.gov web site. Inclusion criteria were being an Emory Healthcare patient with self-reported chronic, distressing levels of pain. Non–English-speaking patients, children, prisoners, and other vulnerable populations were excluded from participating in the study. We assessed 132 potential participants for eligibility. Fifty-five potential participants were excluded from the study (50 declined to participate, with the most common reasons stated as they were not interested or did not have time to participate. 5 were not able to complete online questionnaires or did not live in the area). A total of 74 participants were consented and randomized. Participants were heterogeneous with respect to the cause and type of chronic pain. All participants presented with chronic pain as their primary symptom, had chronic pain clinically determined by providers who are pain fellowship-trained anesthesiologists, and were reporting chronic pain for 3 or more months and associated distress.^31^

### 2.2. Study design

This randomized, wait-list–controlled study was approved by the institutional review board and conducted from the summer of 2018 to the fall of 2019. The study was preregistered as a clinical trial (NCT03495726). The primary outcomes were pain severity and pain catastrophizing. Social and physical functioning were secondary outcomes of interest. Self-reported opioid misuse among the subset of participants using opioids was another preregistered primary outcome. We will report self-reported opioid misuse in a future manuscript because all participants were included in the current report regardless of whether they were currently using opioids. A priori power analysis was not conducted given that this was a pilot study.

Written and informed consent was obtained before any study measures. After consent, participants completed a time 1 prerandomization assessment delivered electronically through Qualtrics. The time 1 assessment contained the following surveys (each described in more detail further): demographics, interest in meditation, pain severity, pain catastrophizing, and social and physical functioning. After the time 1 assessment was completed, each participant was randomized to either a wait-list control group or to a group that received a mindfulness meditation mobile app. We used a random number generator function in excel to randomize participants to each group, and study personnel were blind to group status during all assessments. Participants in the mindfulness group were provided with a subscription code to the meditation app, Headspace (https://www.headspace.com/), as well as written instructions regarding app download and use. Participants were instructed to use the app an average of 10 min/day during the 6-week study period. At the completion of the study, participants completed a time 2 postintervention assessment that included all psychometric measures used at the time 1 assessment. The wait-list group was maintained as a control group throughout the 6- week study period and was provided the Headspace app at the conclusion of the study. App usage was collected from Headspace as total number of meditation sessions completed, the type of session completed, and the amount of time the app was used.

### 2.3. Measures

The following psychometric indices were administered at both the time 1 and time 2 assessments:

#### 2.3.1. Demographics

Participants self-reported their relationship status, education level, race, ethnicity, number of children, recent illness, exercise, and medication use. As a proxy for socioeconomic status (SES), we used participants' home street addresses, extracted from the electronic medical record, to determine their county of residence. County percent poverty rates were then obtained from the U.S. Census' Small Area Income and Poverty Estimates state and county estimates for 2018 and linked to each individual participant.

#### 2.3.2. Interest in mindfulness meditation

At time 1, participants rated their interest on a scale of 1 to 7 (1 = “do not agree at all; 4 = “somewhat agree”; and 7 = “completely agree”) in learning meditation techniques to improve management of their pain, stress, personal relationships, and physical and mental health. They also rated the extent to which they participated in the study because they felt pressured.

#### 2.3.3. Pain severity, pain catastrophizing, and daily functioning

Severity of pain was assessed by administering the pain severity subscale of the Brief Pain Inventory, a 9-item inventory that assesses the severity of pain and the impact of pain on daily functions.^8^ The pain severity subscale consists of 4 items that ask the participant to rate their worst, least, and average pain over the past 24 hours as well as their current pain. Ratings are made on a Likert scale from 0 to 10, with 0 reflecting “no pain” and 10 reflecting “pain as bad as you can imagine.” Ratings from all 4 items were summed, with higher scores reflecting greater pain severity.

To assess pain perception, we used the Pain Catastrophizing Scale, a 13-item inventory designed to measure a participant's tendency to catastrophize pain as a single construct comprising 3 elements of pain perception: rumination, magnification, and helplessness.^34,43^ This scale asks participants to reflect on previous painful experiences and rate their perception of pain on a 5-point Likert scale (0 = “not at all; 4 = “all the time”), with a high score reflecting more pain catastrophizing.

Daily function was measured by assessing physical and social function. The Physical Functioning Scale of the 36-item Short-Form Health Survey was administered to determine physical function.^3,17^ The Physical Functioning Scale is a 10-item questionnaire evaluating how a person's health limits them from partaking in activities such as running, lifting heavy objects, walking more than a mile, climbing one flight of stairs, carrying groceries, kneeling or bending, the ability to perform chores, ability to dress self, wash self, and ability to sit on and get up from the toilet. Participants rate the extent to which pain limits them using a 5-point Likert scale (1 = “cannot do”; 5 = “not at all”), such that a high score reflects relatively better physical functioning. Social functioning was measured using the Social Functioning Impact Short Form from version 2 of the Adult Sickle Cell Quality of Life Measurement Information System (ASCQ-Me v2.0)^22^ (https://www.healthmeasures.net/). This 5-item measure was developed to assess the ability to participate in social roles over the past 30 days and measures a participant's dependence on others to take care of their health as well as how often one's health interferes with daily social tasks. Participants rate the extent to which pain limits their social activities using a 5-point Likert scale (1 = “very much”; 5 = “not at all”), such that a high score reflects relatively better social functioning.

### 2.4. App-delivered mindfulness program

Participants randomized to the mindfulness meditation intervention group were provided a 1-year subscription to Headspace, a meditation app that has more than 30 million worldwide downloads.^6,13^ They were provided written instruction and assistance with downloading the app and were given the following directives: “Please try to do one session or at least 10 minutes of meditation every day for 6 weeks. Just do the best you can. If you miss a day, that's OK.” Participants were asked to complete the first level of the introduction or “Basics” program, which comprised 10 sessions that introduce core mindfulness principles and practices. The primary practices in this program are body scans, a guided practice that involves bringing one's attention and awareness to the sensations of various regions of the body, as well as mindfulness meditation that entrains awareness to sensations of the breath. During both body scans and breath awareness practices, participants are instructed to observe the sensations of the breath or body, as well as any thoughts and feelings that arise, without judgement. Participants are instructed that when other thoughts or feelings naturally arise, they can become aware of these thoughts and feelings, acknowledge them, and place their focus back on the sensation that is the target of their practice.

We instructed participants that after completing *The Basics* course, they should complete the *Pain Management* program, which has 3 levels, each of which has 10 sessions (30 sessions total). The Pain Management program not only has similar material and practices as the Basics course but also includes didactic information and meditation aimed at cultivating a nonjudgmental awareness of pain sensations more specifically. Participants practice exploring their pain sensations without judging them as good or bad, to notice when thoughts or feelings arise, and to place their focus back on their sensations in a nonjudgmental way. App usage data were acquired from Headspace and quantified as minutes spent meditating as well as number of modules used. The wait-list control group received information about the app at the end of the 6-week study period.

### 2.5. Statistical analysis

Missing items were imputed using expectation maximization (missing items never accounted for more than 5% of total data) using other items within each scale as predictor variables. Self-report scores were calculated according to each measures' recommended method of calculating sum scores. All analyses were conducted using IBM SPSS Statistics v26 for Windows, with a significance level set at *P* < 0.05. We used the Shapiro–Wilk test to assess data normality. The following variables had a significant Shapiro–Wilk test, indicating a nonnormal distribution: poverty, social functioning at times 1 and 2, and time 2 pain catastrophizing. We used nonparametric testing to evaluate these variables and outcomes.

We generated descriptive statistics (mean, median, and SD) for all demographic variables (age, gender, area-level SES, religious affiliation, relationship status, education, and race) and evaluated randomization success using Pearson χ^2^ (for categorical variables) and independent *t* tests or Mann–Whitney *U* tests (for continuous variables). To evaluate the feasibility of using the app-delivered mindfulness program (aim 1), we quantified the percentage of participants who used the app at least one time, the percentage of participants who used a meditation from the pain management course, the percentage of participants who used the app at least half of the recommended amount (210 minutes or more), and the percentage of participants who used the app for the recommended amount (420 minutes or more). Adherence was defined as practicing at least half the recommended amount.

Our second aim was to identify characteristics of participants who adhered to the app-delivered mindfulness intervention, as well as those who did not use the app at all. We conducted independent samples *t* tests to examine participants randomized to the mindfulness meditation group who (1) did not try the app and (2) adhered to the intervention differed on any of the continuous variables (interest, pain catastrophizing, age, and area-level SES). We conducted χ^2^ tests to evaluate whether either lack of trying or adherence were significantly more likely within the categorical variables of gender, education level, relationship status, religious affiliation, and race/ethnicity.

To evaluate changes in pain severity, pain catastrophizing, and general functioning among participants randomized to Headspace, compared with those patients randomized to the wait-list control group (aim 3), we examined group by time interactions by calculating repeated measures analysis of variance. Paired *t* tests and Wilcoxon signed-rank tests were used to assess within-group changes in all measures. Independent *t* tests and Mann–Whitney *U* tests were used to evaluate group differences at time 2. Finally, to evaluate whether changes in self-reported outcomes were related to mindfulness practice time, we conducted the Spearman rho correlation between app use and any self-reported variables for which there was a significant main effect within the mindfulness group or significant group by time interaction.

## 3. Results

Of the 77 participants consented, 3 participants dropped out before completing the time 1 assessment surveys. Participants (n = 74; 47 [64%] female, 27 [36%] male) were between the ages of 23 to 89 years (Table 1). Twenty-one participants were lost to follow-up before completing the time 2 assessment (7 in the mindfulness group, 14 in the wait-list group). There were no serious adverse events reported in either group and no known harms involved with the intervention group.

**Table 1:** Demographic characteristics according to group randomization.

	M (n = 33)	W (n = 41)	Significance
Age	51.76 (14.1)	54.15 (15.3)	0.492
Area-level SES	12.64 (3.31)	12.13 (4.13)	0.568
Gender	22 female	25 female	0.765
Religious affiliation	16 yes	18 yes	0.694
Relationship status			0.357
Single	9	11	
Divorced	3	10	
Living with someone	1	0	
Living with partner	3	2	
Married	17	18	
Education			0.518
High school degree	3	7	
Some college, no degree	6	11	
Associate degree	4	6	
Bachelor's degree	12	6	
Master's degree	5	7	
Professional degree	1	1	
Doctorate degree	2	3	
Race			
White	18	21	0.939
Black or African American	14	19	
Other	1	1	

### 3.1. Feasibility of app usage

Before randomization, most participants (99%) reported a high level of interest in using the app, and a desire to manage pain was the most highly endorsed reason for wanting to practice mindfulness meditation (96%). Although only one participant reported a lack of interest in using the app, 17 (23%) reported joining the study because they felt pressured or believed they “were supposed to.” Of the participants randomized to the app group, 28 (85%) successfully downloaded the app and used it at least one time. Five participants randomized to the mindfulness group did not use the app at all. Sixteen (48.5%) participants used at least one meditation from the *Pain Management* course. Fourteen (42%) participants adhered to the intervention, using the app for at least half the recommended time (210 minutes), and 4 participants (12%) used the app for the recommended amount of time (420 minutes) or more.

### 3.2. Characteristics related to app usage

**Figure 2.**
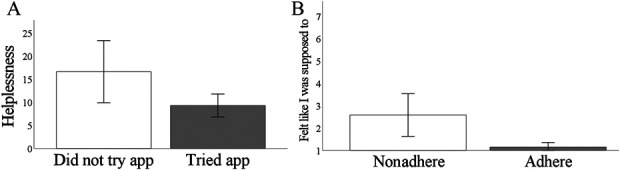
(A) Patients who used the app at least one time had lower self-reported helplessness scores than patients who did not try the app. (B) Adherent patients (those who used the app for at least 210 minutes or at least half the recommended time) reported feeling less pressured to join the study than those who did not adhere.

Independent samples *t* tests revealed that participants who were randomized to the mindfulness intervention group but did not engage in any meditation practice had higher self-reported pain catastrophizing. Specifically, these participants scored higher on the helplessness subscale of the pain catastrophizing scale before randomization (t(31) = 6.197, *P* = 0.02, d = 1.16) (Fig. 2A). χ^2^ tests indicated that participants with less than a college degree were more likely not to use the app at all (χ^2^ (6, N = 33) = 16.41, *P* = 0.012). Not trying the app was not significantly related to any other interest, severity, or catastrophizing variable, nor was it significantly associated with gender, relationship status, affiliation with a religious group, or race/ethnicity. Independent samples and Mann–Whitney *U* tests indicated that participants who were randomized to the mindfulness group and adhered to the intervention disagreed with the statement, “I am participating in this study because I felt like I was supposed to.” (Z = −2.50, *P* = 0.012, r = 0.43) (Fig. 2B). No other variables including pain severity or pain catastrophizing variables were significantly related to adherence, nor were age or SES significantly related to adherence. χ^2^ tests indicated that adherence was not significantly associated with gender, relationship status, education, affiliation with a religious group, or race/ethnicity.

### 3.3. Effects of group randomization

Independent samples *t* tests and Mann–Whiney *U* tests indicated that there were no significant differences between the groups at the time 1 assessment, except for pain severity. Because of chance, participants randomized to mindfulness had significantly lower pain severity ratings than those randomized to the wait-list control group (t(72)—2.01, *P* = 0.048) (Table 2). For this reason, we controlled for pain severity in all subsequent analyses by entering it as a covariate in all repeated measures analyses.

**Table 2:** Mean and SD for symptom scores by group assignment and time point.

Outcome	Time 1 (preintervention)			Time 2 (postintervention)		
	Mind	WL	Significance	Mind	WL	Significance
Pain severity	**22.1 (9.90)**	**26.6 (9.58)**	**0.048**	**21.0 (9.87)**	**27.5 (9.59)**	**0.020**
Pain catastrophizing	24.7 (14.7)	25.2 (11.2)	0.873	18.8 (14.7)	19.5 (12.8)	0.707
Rumination	8.61 (5.56)	9.14 (4.33)	0.647	8.09 (6.60)	8.578 (5.76)	0.673
Magnification	5.70 (3.49)	4.934 (3.08)	0.328	4.54 (3.37)	3.93 (3.11)	0.629
Helplessness	10.4 (6.74)	10.8 (5.54)	0.796	7.47 (6.68)	8.62 (6.12)	0.336
Physical functioning	39.4 (8.78)	38.5 (8.48)	0.643	37.2 (5.86)	37.1 (6.25)	0.914
Social functioning	14.1 (5.85)	14.67 (6.25)	0.683	15.2 (6.41)	13.5 (6.35)	0.392

Wilcoxon signed-rank and paired *t* tests indicated that participants randomized to the mindfulness group reported a significant decrease in pain catastrophizing (Z = −3.14, *P* = 0.002, r = 0.61) (Table 3). Participants reported reductions in all 3 subscales of the pain catastrophizing scale; however, only the helplessness subscale reached significance: rumination (Z = −1.41, *P* = 0.16), magnification (Z = − 1.80, *P* = 0.07), and helplessness (Z = −2.88, *P* = 0.004, r = 0.57). There was also a significant degradation in physical functioning (t(23) = 3.74, *P* = 0.001, d = 0.76) in the mindfulness group. Among participants randomized to wait-list, there was a significant degradation in physical (t(25) = 2.35, *P* = 0.03, r = 0.46) and social (Z = 2.15, *P* = 0.03, r = 0.41) functioning. There were no other significant effects of time in either group.

**Table 3:** Results of within-group effects of time and group by time interaction effects.

Outcome	Mindfulness				Wait-list				Group by time interaction	
	Time 1 (pre)	Time 2 (post)	t/Z	Sig.	Time 1 (pre)	Time 2 (post)	t/Z	Sig.	F	Sig.
Pain severity	22.1 (9.90)	21.0 (9.87)	0.69	0.495	26.6 (9.58)	27.5 (9.59)	0.80	0.429	1.11	0.298
Pain catastrophizing	24.7 (14.7)	18.8 (14.7)	**−3.14**	**0.002**	25.2 (11.2)	19.5 (12.8)	−1.83	0.067	0.41	0.524
Rumination	8.61 (5.56)	8.09 (6.60)	−1.41	0.160	9.14 (4.33)	8.578 (5.76)	−0.152	0.879	0.13	0.721
Magnification	5.70 (3.49)	4.54 (3.37)	−1.80	0.072	4.934 (3.08)	3.93 (3.11)	−1.39	0.165	0.7	0.409
Helplessness	10.4 (6.74)	7.47 (6.68)	**−2.88**	**0.004**	10.8 (5.54)	8.62 (6.12)	−1.59	0.111	0.41	0.526
Physical functioning	39.4 (8.78)	37.2 (5.86)	**−3.24**	**0.001**	38.5 (8.48)	37.1 (6.25)	**−2.36**	**0.019**	0.08	0.785
Social functioning	14.1 (5.85)	15.2 (6.41)	−1.24	0.215	14.67 (6.25)	13.5 (6.35)	**−2.15**	**0.032**	**4.72**	**0.035**

Repeated measures analysis of variance, controlling for time 1 pain severity, indicated that there was a group by time interaction effect for social functioning, such that participants randomized to mindfulness reported improved social functioning compared with participants randomized to the wait-list group (F(50) = 4.72, *P* = 0.035, ${\text{η}}_{\text{p}}^{2}$ = 0.09). There was not a significant group by time interaction for any other outcome (pain severity: F(50) = 1.11, *P* = 0.298; pain catastrophizing: F(48) = 0.41, *P* = 0.524; physical functioning: F(47) = 0.08, *P* = 0.785). Independent *t* tests and Mann–Whiney *U* tests indicated that there were no significant differences between the groups at time 2, except for pain severity, which remained significantly different at time 2 (t(50) = 2.40, *P* = 0.020). Finally, app usage was not significantly correlated with changes in any outcome measures among participants randomized to Headspace.

## 4. Discussion

Existing research indicates that mindfulness meditation practice can be effective for participants with chronic pain and affects their level of anxiety, coping, and quality of life.^7,37^ Although some studies have shown that the practice of mindfulness meditation affects ratings of pain intensity and unpleasantness,^51^ other studies have found that mindfulness practice is beneficial primarily through a wider array of “nonspecific” effects. Patients have been shown to have a reduction in rumination and pain catastrophizing and an increase in self-efficacy or acceptance of pain symptoms (reviewed in Ref. 36). Some researchers have theorized that decreases in reported pain intensity may be an early and relatively acute outcome of mindfulness practice or induction resulting from changes in the affective or evaluative components of pain, whereas long-term meditation practice may lead to a decoupling of the somatosensory and appraisal processes.^40,51^ The latter effect would essentially unyoke the sensory experience from the meaning of the pain, which may be of particular importance for patients living with chronic pain, for whom the emphasis is often on achieving quality of life despite the pain. We found that patients living with chronic pain who were randomized to the app-delivered mindfulness group, compared with those randomized to a wait-list control group, reported improvements in social functioning with a medium effect size. This, despite a deterioration in physical functioning, suggests that mindfulness may allow patients to remain engaged with their social lives despite their pain. In addition, within the mindfulness group, patients reported reductions in pain catastrophizing, specifically in the helplessness aspect of catastrophizing.

Although we refer to the mindfulness program used here as an *intervention* in as much as it was a manipulation of the participant's environment for the purpose of modifying health-relevant processes and/or end points,^18^ Headspace is a for-profit app in the consumer domain that is used by many diverse clinical and nonclinical populations. It is clear that people meditate for many and varied reasons and with a vast array of goals and expected benefits.^24^ In addition to examining the effectiveness of app-delivered mindfulness, we were interested in participants' self-reported interest in using the app and how their interest was related to their subsequent engagement with the app. Although we did not find that interest levels predicted adherence, participants who did not adhere were more likely to endorse feeling pressured to enroll in the study. Of the participants who practiced at least half of the recommended amount, no one endorsed feeling pressured to enroll in the study. By contrast, 5 participants (26%) who did not adhere reported feeling as though they “were supposed to enroll” before randomization. Moreover, we found that those who did not try the app scored higher on the helplessness subscale of the pain catastrophizing measure before randomization. In other words, participants' baseline levels of pain catastrophizing predicted their subsequent app usage. Together, these findings highlight the importance of participant motivation and self-efficacy and suggest that the success of MBIs in clinical contexts will be affected by whether patients feel intrinsically motivated to engage with the program. This finding is consistent with previous research using app-delivered mindfulness meditation,^27^ and it will be important in future research to examine whether approaches to increase intrinsic motivation and self-efficacy, such as motivational interviewing, influence the outcomes of MBIs for patients with chronic pain.^41^

We chose to use the Headspace app for 2 reasons. First, at the commencement of the study it held the highest Mobile Application Rating Scale^42^ rating of all available mindfulness apps.^26^ Second, Headspace was one of the few commercially available apps with content that specifically delivered mindfulness meditation targeting pain. However, the number and diversity of apps that deliver mindfulness content is growing and understanding who uses and benefits from app-delivered mindfulness is important. Although there is little research thus far on predictors of mindfulness app usage, there is an emerging body of research indicating that individual differences and sociodemographic factors affect mindfulness practice.^24^ For example, National Health Interview Survey data show that education level, race/ethnicity, and sex/gender (here we refer to sex/gender differences to indicate agnosticism over whether differences in mindfulness engagement between men and women reflect biologically or socially influenced differences or a complex combination) predict engagement with mindfulness practices.^33^ Within the context of clinical trials, a sex/gender difference was found in a recent meta-analysis of randomized controlled trials investigating the effectiveness of MBIs.^2^ When mindfulness interventions are explicitly targeted for minority populations, they are primarily offered to children and adolescents.^10,45^ These epidemiological data indicate that white, female, and highly educated individuals are more likely to engage in mindfulness practices and more likely to enroll in clinical trials of mindfulness interventions. Somewhat consistent with existing research, we found that participants randomized to the mindfulness group who were less educated were more likely not to try the app at all. This is in line with some previous research indicating that mindfulness app users are more likely to have a college education,^38^ and identifying ways to remove this barrier to mindfulness use will be crucial toward increasing access for patients who may benefit for managing their chronic pain. Although there was no effect of gender on adherence or engagement, women outnumbered men in enrolling in the study almost 2:1.

It is worth noting that in contrast to our predictions, several demographic factors were not significantly related to app use or adherence. We found that engagement with the app was not related to area-level poverty, gender, or race/ethnicity. Area-level poverty rates have been used as an indicator for other social determinants of health and barriers to stable health care access.^4^ Studies have shown that individuals residing in areas with >10% poverty rate have less utilization of preventive colorectal cancer screening.^23^ Increasing area-level poverty rates are associated with decreased utilization of mammograms, clinical breast examinations, colonoscopies, sigmoidoscopies, and fecal occult blood tests.^39^ In this study, all enrollees reported having access to a smartphone or other device that could be used to download the app. It is likely that more extreme levels of poverty, such as not having access to a smart phone, would have been a bigger barrier for participants.

Although participants randomized to app usage reported significant improvements in the helplessness aspect of pain catastrophizing, as well as significant improvements in social functioning compared with the wait-list group, none of these effects were correlated with practice time. This is consistent with a recent study that examined the impact of mindfulness delivered by the same app for patients with chronic pain, which also found that self-reported changes were not related to app usage.^46^ A recent meta-analysis found that the efficacy of MBIs for patients with chronic pain did not differ by length or frequency of intervention or by type of MBI.^19^ It may be that app-delivered practice time is less predictive of outcomes than is practice for in-person or group mindfulness interventions. Alternatively, the effects observed in this study may be explained by some nonspecific aspect of app use. A recent study compared the effects of mindfulness meditation delivered through Headspace with a sham meditation condition. Although participants in both groups improved on measures of cognition (self-reported mindfulness, executive function, and critical thinking), mindfulness did not confer more benefit than the sham meditation.^32^ Our study was limited in not having an active control condition, and we may have found that a sham meditation had similar effects on self-reported pain processing. Related, the effects observed here may have been influenced by demand characteristics or by the placebo effect, especially given that the goal of pain management was made clear and reinforced in the title and content of the meditations. There has been extensive thought on the use of app-delivered mindfulness, especially with respect to whether it contains the key elements of an MBI,^9^ and future research on the “active ingredients” of app-delivered interventions will be critical.

### 4.1. Limitations

Although this pilot study may have been underpowered to detect small effects, it is likely that it was large enough to accurately estimate the effect size for the outcome measures that we interrogated.^44^ Moreover, we recruited a heterogeneous patient population presenting in a chronic pain clinic, and it is possible that subgroups of participants may have experienced more or less benefit from the mindfulness app. Lending support to this possibility, there is some evidence that mindfulness has differential effects depending on the type of chronic pain.^7,37^ However, the evidence is mixed, and another recent meta-analysis found that the efficacy of MBIs did not differ by medical condition.^19^ In addition, we chose not to include a mindfulness self-report measure to minimize the burden on study participants, and for this reason, we are unable to examine whether self-reported mindfulness was affected by meditation practice or related to the changes in pain outcomes. Here, we report the immediate effects of the mindfulness app, but it is unclear whether the observed changes endured beyond the immediate practice period. This study adds to what is known about the use and effectiveness of app-delivered mindfulness, but there is a great need for more research in this area.

## Disclosures

The authors have no conflicts of interest to declare.
